# Bronchial carcinoid in a 39-year-old man treated for bronchial asthma: a case report

**DOI:** 10.1186/1757-1626-2-7414

**Published:** 2008-12-16

**Authors:** Justyna Emeryk, Elżbieta Czekajska-Chehab, Elżbieta Korobowicz, Marta Korbel, Irena Węgrzyn-Szkutnik, Janusz Milanowski

**Affiliations:** 1 Chair and Department of Pulmonology, Oncology and Allergology, Medical University of Lublin, Jaczewski Street 8, 20-950 Lublin, Poland; 2 First Department of Radiology, Medical University of Lublin, Jaczewski Street 8, 20-950 Lublin, Poland; 3 Chair and Department of Clinical Pathomorphology, Medical University of Lublin, Jaczewski Street 8, 20-950 Lublin, Poland

## Abstract

A case study of 39-year old man with persistent wheezing, episodes of haemoptysis and dry cough unsuccessfully treated with inhaled beta2-agonists and steroids for about 10 months. Chest radiograph revealed a disproportion in dimensions between both lungs, with the left one being smaller than the right one. Spirometry demonstrated a restrictive pattern. During bronchoscopy, a polypoid endobronchial tumor, localized in the left main bronchus, completely occluding its lumen, was found. The tumor was diagnosed as carcinoid. In this case, due to the lack of characteristic symptoms, diagnosis of carcinoid was delayed. Patients unsuccessfully treated for bronchial asthma or chronic obstructive pulmonary disease should undergo bronchoscopic examination.

## Case presentation

**Figure 1:**
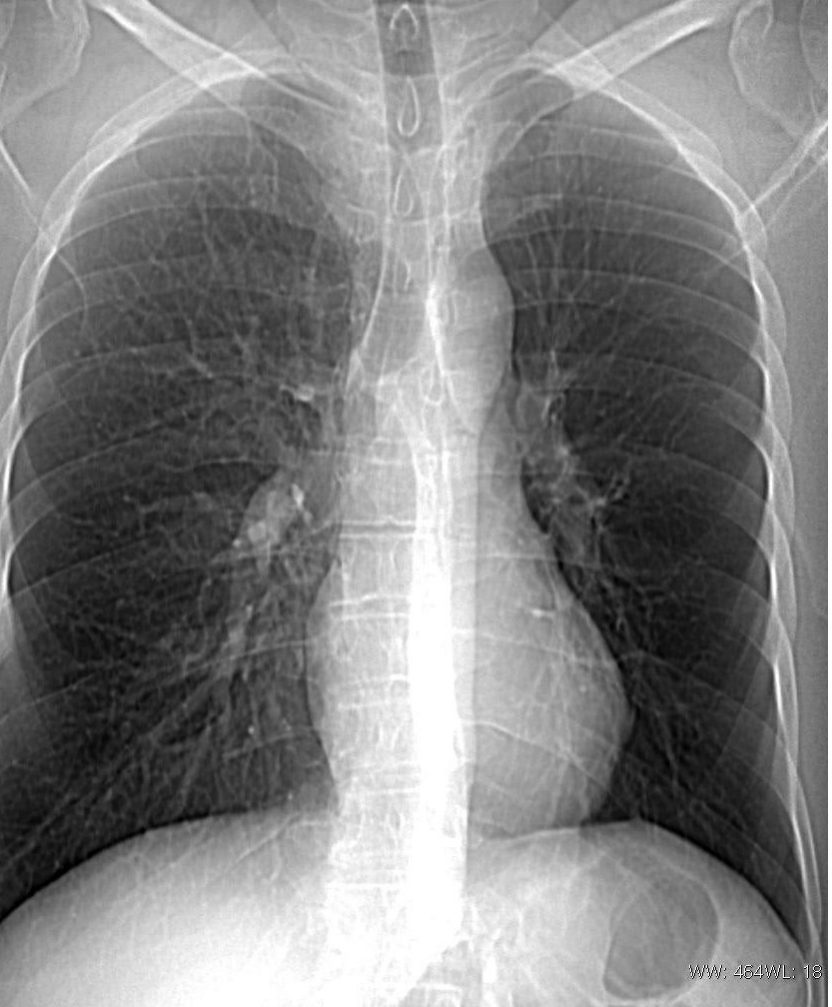
**Chest radiograph performed at admission to the hospital**.

A 39-year-old white man, working as a house builder, was admitted to our pulmonary ward in August 2007 with an eleven-month history of persistent wheezing, heard not only during auscultation but also by patient himself. This wheezing had no correlation with physical exertion. He also reported a few episodes of haemoptysis within last 7 months and dry cough for about a month. Patient's weight was 81.5 kg, his height was 180 cm. Except from nodular goitre (in euthyreosis), the patient reported no other complaints or diseases in his medical history. His family history was not clinically significant. He had a 10 pack-years smoking history, until the age of 31 when he stopped smoking. He drank alcohol occasionally. His symptoms were unsuccessfully treated with inhaled β_2_-agonists and steroids for about 10 months. Apart from these medications, he took no other drugs. On auscultation, there were wheezes in upper fields of both lungs. The chest radiograph (figure 1) showed a disproportion in dimensions and vascular markings between both lungs- the left one was significantly smaller and had decreased vascular markings in comparison to the right one- these findings might be the expression of left lung hypoplasia. The heart silhouette was discretely displaced to the left side.

**Figure 2:**
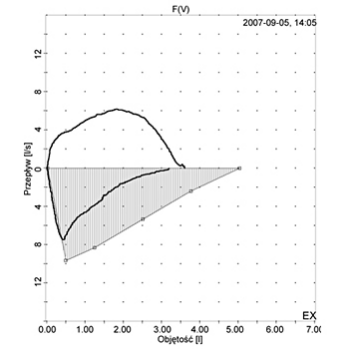
**Flow-volume curve showing a restrictive pattern performed at admission to the hospital**.

Spirometry performed during hospitalization in our clinic showed a restrictive pattern (figure 2) with forced expiratory volume in one second (FEV_1_) of 59% predicted and forced vital capacity (FVC) of 66% predicted. The FEV_1_/FVC ratio was 70% predicted.

Bronchoscopy was performed, revealing a polypoid endobronchial tumor, localized in the left main bronchus (2 cm from tracheal bifurcation), completely occluding its lumen. The tumor was biopsied and the specimen was sent to histological examination which gave the diagnosis of bronchial carcinoid.

**Figure 3:**
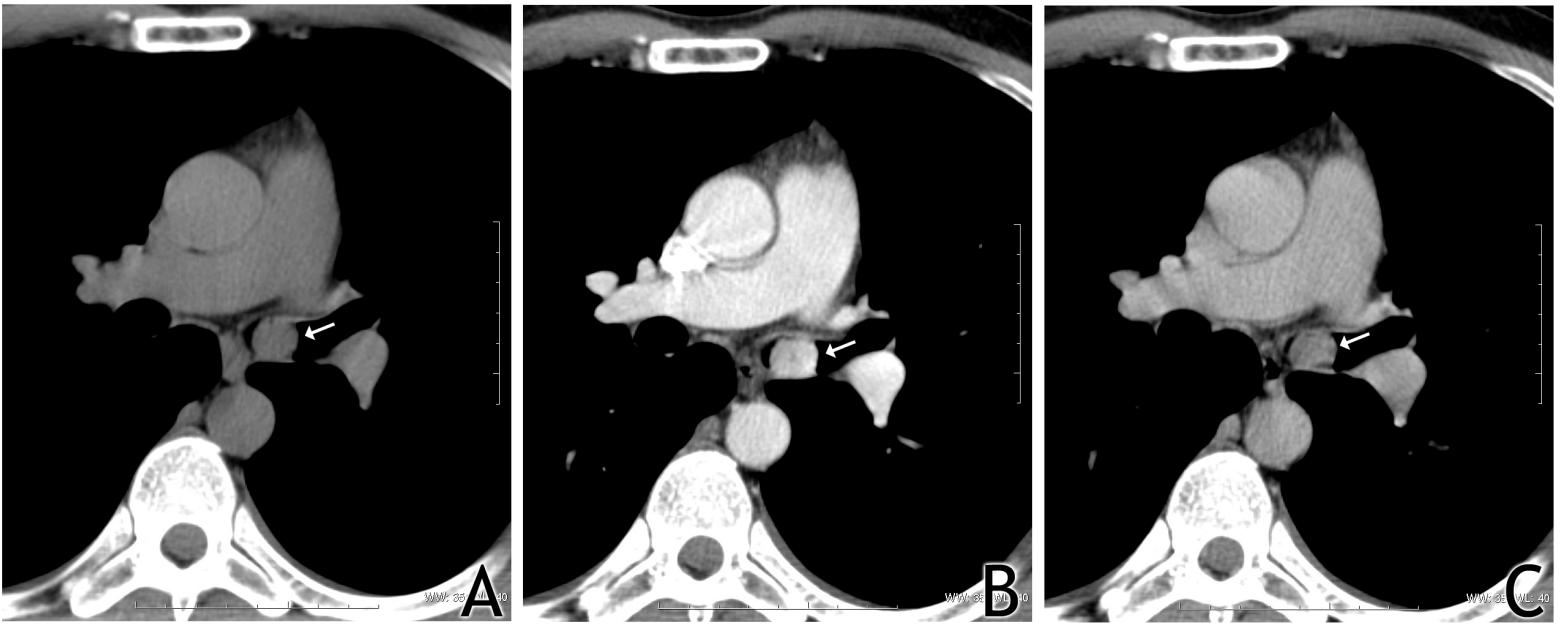
**High enhancement of the tumor (arrow) after contrast medium administration: on the left- before administration of contrast medium, in the middle-after administration; arterial phase, on the right-parenchymal phase**.

Thoracic computed tomography showed a polypoid mass in the left main bronchus with a diameter of about 20 mm. The tumor showed very high enhancement after contrast medium administration: from 9 HU (Hounsfield units) (figure 3a) to 119 HU in arterial phase (figure 3b) and 47 HU in parenchymal phase (figure 3c), which is characteristic for carcinoid.

**Figure 4:**
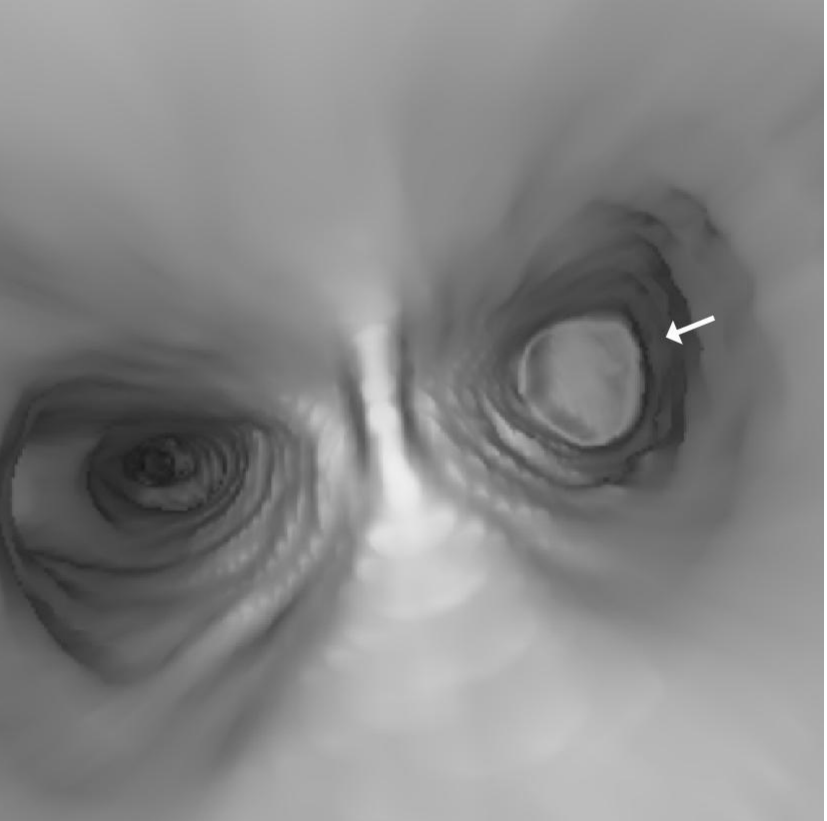
**Total occlusion of the left main bronchus by the tumor (arrow)**.

**Figure 5:**
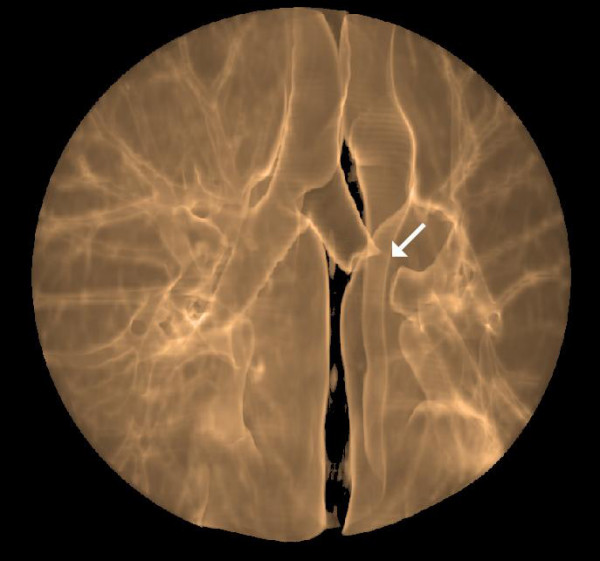
**Amputation of the left main bronchus by the tumor (arrow)**.

Hilar and mediastinal nodes were not enlarged. In virtual bronchoscopy, as well as in transparent reconstruction of the airways, a total occlusion of the left main bronchus by the tumor could be observed (Figure 4 and 5).

The patient was transferred to thoracic surgery ward for surgical treatment. He underwent left pulmonectomy and histopathological analysis of post-operative material confirmed the diagnosis of bronchial carcinoid. There were no complications during post-operative period and the patient was discharged from hospital 10 days after the operation. Then, the patient went abroad and was lost for follow-up until May 2008. On control visit in May, he was in very good condition, presenting no signs of pulmonary disease. On physical examination, except from diminished breath sounds and dullness to percussion over left lung (due to pulmonectomy and consequent pleural fluid accumulation) no abnormalities were found. In control computed tomography there were no signs of recurrence of the disease.

## Discussion

Pulmonary carcinoids comprise 1-2% of all lung tumors. They may develop in many locations in the body but most often, they are found in small intestine (26%), respiratory system (25%) and appendix (19%) [1].

Carcinoid tumors are classified as typical or atypical according to histopathological criteria. This division has a strict extrapolation to survival rates: in case of typical pulmonary carcinoids 5-year survival rate is over 90%, in atypical ones- it is within the range of 40-60% [2].

The following symptoms are observed most often in patients with carcinoid localized in respiratory system: haemoptysis, cough, recurrent pulmonary infections, fever, chest discomfort, unilateral wheezing and shortness of breath [3].

Due to the lack of characteristic symptoms, diagnosis of pulmonary carcinoid is delayed and patients are often misdiagnosed with asthma or chronic obstructive pulmonary disease. According to three studies, there is an average delay of, respectively, 19, 13 and 10 months from the first symptoms to the final diagnosis of carcinoid [4-6]. Although general prognosis for patients with this neoplasm is quite favorable, it is obvious that the earlier diagnosis is made, the chances for radical treatment increase.

As the majority of pulmonary carcinoids (70%) are located in the main or lobar bronchi [7], they are within the reach of a bronchoscope. According to British Thoracic Society, flexible bronchoscopy is a safe procedure and there are no controlled studies concerning the factors disqualifying a patient from it [8]. Bronchoscopy cannot replace computed tomography, these both procedures are complementary, but to be able to orientate the treatment and prognosis, we have to know the histopathological diagnosis and the specimens for examination can be easily and safely provided by bronchoscopy.

## Conclusion

This case is a good example of misdiagnosis of the disease. Extended clinical diagnosis, including computed tomography and bronchoscopy, should be considered in all cases of bronchial asthma or chronic obstructive pulmonary disease which do not respond to standard treatment.

## Consent

"Written informed consent was obtained from the patient for publication of this case report and accompanying images. A copy of the written consent is available for review by the Editor-in-Chief of this journal."

## Competing interests

The authors declare that they have no competing interests.

## Authors' contributions

JE performed acquisition of data, review of literature and wrote the paper. ECC performed computed tomography imagings and prepared virtual bronchoscopy reconstructions. EK performed histopathological examination of the tumour. MK was responsible for patient care, follow-up and data collection. IWS was responsible for patient care and drafting of paper. JM performed acquisition of data, revised the manuscript and provided general support as the head of department. All authors read and approved the final manuscript.

**Figure 6:**
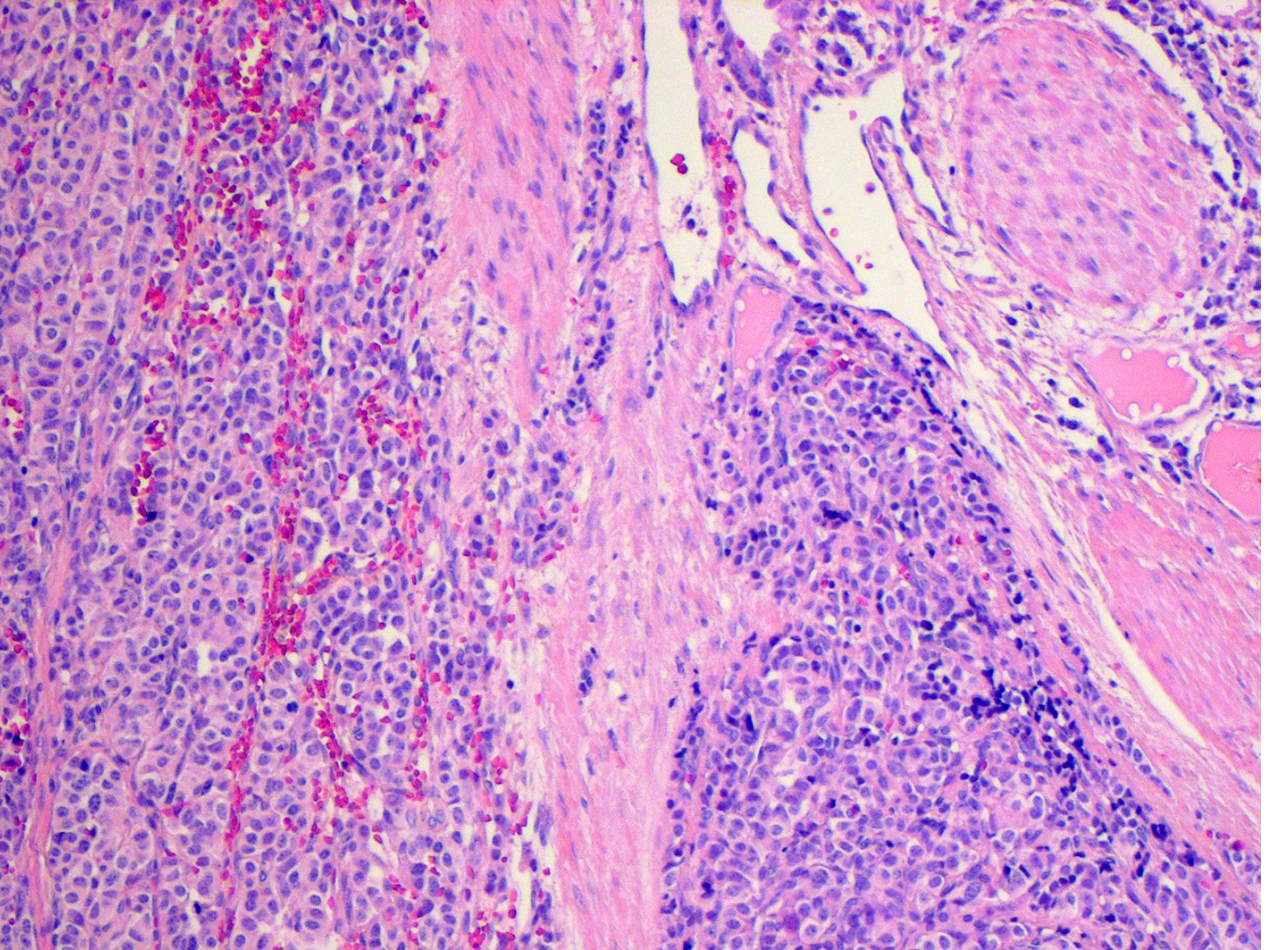
**Microscopic imaging of bronchial carcinoid (hematoxylin and eosin staining)**.

**Figure 7:**
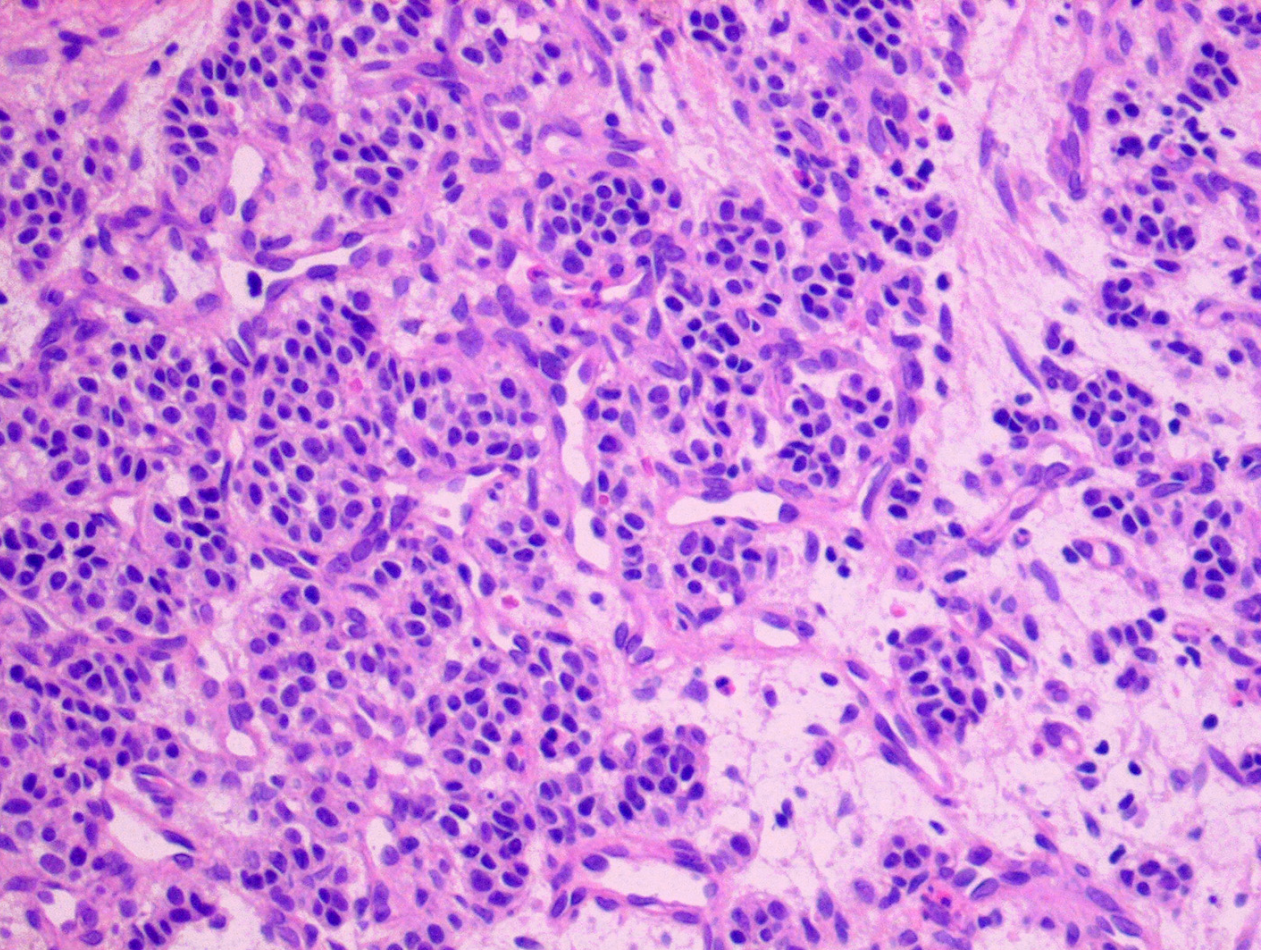
**Microscopic imaging of bronchial carcinoid (hematoxylin and eosin staining)**.

**Figure 8:**
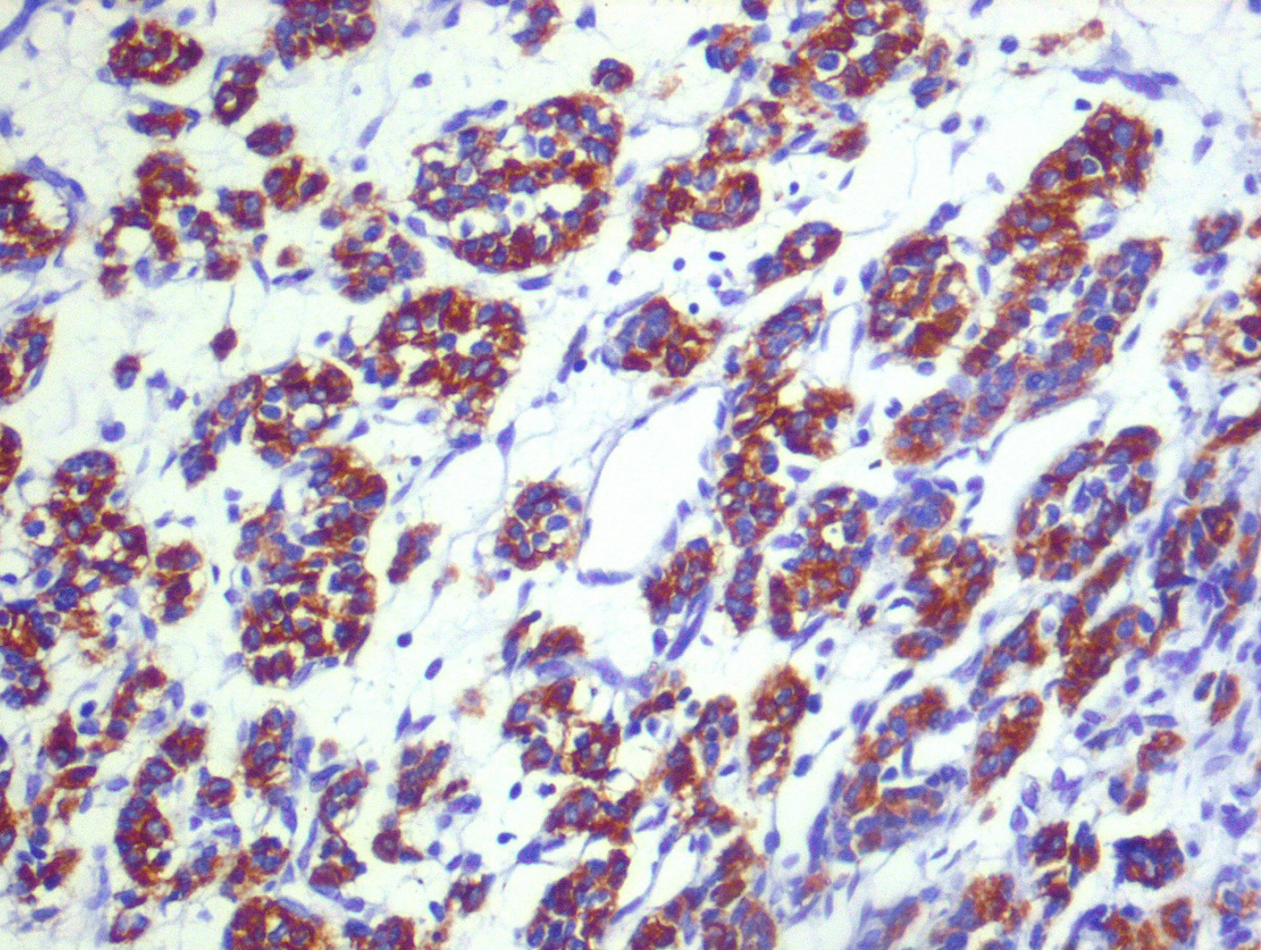
**Immunohistochemical staining positive for chromogranine A**.

**Figure 9:**
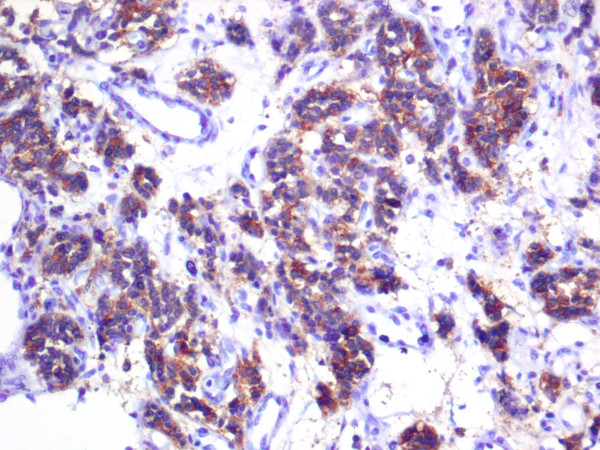
**Immunohistochemical staining positive for synaptophysin**.
